# Supplementary material for: Reaching underserved South Africans with integrated chronic disease screening and mobile HIV counselling and testing: A retrospective, longitudinal study conducted in Cape Town
Source: PLoS One. 2021 May 4;16(5):e0249600. doi: 10.1371/journal.pone.0249600 (PMC8096085; doi:10.1371/journal.pone.0249600)
Supplement: S1 File — (DOCX) [file pone.0249600.s001.docx]

**Table of studies conducted at the mobile clinic**

| **Study authors** | **Recruitment method** |
| --- | --- |
| (Bassett et al., 2014) | This was a cost effectiveness study that did not recruit participants. |
| (Govindasamy et al., 2011) | The mobile registered self-presenting patients at the mobile, who after receiving a referral to their preferred clinic, were followed up to track linkage-to-care. |
| (Kranzer et al., 2011) | A random sample of adults in a high HIV disease burden community were invited to test at the mobile to assess predictors of HIV testing and HIV yield at the mobile clinic. |
| (Kranzer et al., 2012) | Self-presenting HIV negative patients with tuberculosis (TB) symptoms and self-presenting HIV positive patients were invited to participate in a study investigating the feasibility, yield, and cost-effectiveness of active TB case-finding at a mobile clinic. |
| (Kranzer et al., 2012) | This study randomly invited 1300 residents from a high HIV disease burden, peri-urban community to take up incentivised HIV testing and compared HIV positivity with a sample of patients who had self presented at the mobile clinic during the same period. |
| (Maughan-Brown et al., 2018) | This study invited patients who had self-presented at the mobile clinic, who had tested HIV positive, and who were eligible for ART to participate in a randomised control trial of an cash incentive for linkage to care. |
| (Smith et al., 2016) | Self‐presenting adolescents and young adults were invited to participate in a study investigating the fidelity, usability and acceptability of the AtomoRapid HIV Rapid self‐testing device. |
| (van Schaik et al., 2010) | The mobile registered self-presenting patients at the mobile clinic to assess the age and gender differences between patients accessing the mobile clinic and those accessing a clinic facility and hospital facility for HIV testing. |

**References**

Bassett, I. V., Govindasamy, D., Erlwanger, A. S., Hyle, E. P., Kranzer, K., van Schaik, N., Noubary, F., Paltiel, A. D., Wood, R., Walensky, R. P., Losina, E., Bekker, L.-G., & Freedberg, K. A. (2014). Mobile HIV Screening in Cape Town, South Africa: Clinical Impact, Cost and Cost-Effectiveness. *PLoS ONE*, *9*(1). https://doi.org/10.1371/journal.pone.0085197

Govindasamy, D., van Schaik, N., Kranzer, K., Wood, R., Mathews, C., & Bekker, L.-G. (2011). Linkage to HIV Care From a Mobile Testing Unit in South Africa by Different CD4 Count Strata. *JAIDS Journal of Acquired Immune Deficiency Syndromes*, *58*(3), 344–352. https://doi.org/10.1097/QAI.0b013e31822e0c4c

Kranzer, K, Govindasamy, D., van Schaik, N., Thebus, E., Davies, N., Zimmermann, M., Jeneker, S., Lawn, S., Wood, R., & Bekker, L.-G. (2012). Incentivized recruitment of a population sample to a mobile HIV testing service increases the yield of newly diagnosed cases, including those in need of antiretroviral therapy. *HIV Medicine*, *13*(2), 132–137. https://doi.org/10.1111/j.1468-1293.2011.00947.x

Kranzer, Katharina, Lawn, S. D., Meyer-Rath, G., Vassall, A., Raditlhalo, E., Govindasamy, D., van Schaik, N., Wood, R., & Bekker, L.-G. (2012). Feasibility, Yield, and Cost of Active Tuberculosis Case Finding Linked to a Mobile HIV Service in Cape Town, South Africa: A Cross-sectional Study. *PLoS Med*, *9*(8), e1001281. https://doi.org/10.1371/journal.pmed.1001281

Kranzer, Katharina, van Schaik, N., Karmue, U., Middelkoop, K., Sebastian, E., Lawn, S. D., Wood, R., & Bekker, L.-G. (2011). High Prevalence of Self-Reported Undiagnosed HIV despite High Coverage of HIV Testing: A Cross-Sectional Population Based Sero-Survey in South Africa. *PLoS ONE*, *6*(9), e25244. https://doi.org/10.1371/journal.pone.0025244

Maughan-Brown, B., Smith, P., Kuo, C., Harrison, A., Lurie, M. N., Bekker, L.-G., & Galárraga, O. (2018). A Conditional Economic Incentive Fails to Improve Linkage to Care and Antiretroviral Therapy Initiation Among HIV-Positive Adults in Cape Town, South Africa. *AIDS Patient Care and STDs*, *32*(2), 70–78. https://doi.org/10.1089/apc.2017.0238

Smith, P., Wallace, M., & Bekker, L.-G. (2016). Adolescents’ experience of a rapid HIV self-testing device in youth-friendly clinic settings in Cape Town South Africa: A cross-sectional community based usability study. *Journal of the International AIDS Society*, *19*(1). https://doi.org/10.7448/ias.19.1.21111

van Schaik, N., Kranzer, K., Wood, R., & Bekker, L.-G. (2010). Earlier HIV diagnosis—Are mobile services the answer? *South African Medical Journal = Suid-Afrikaanse Tydskrif Vir Geneeskunde*, *100*(10), 671–674.
